# Protocol for expanded indications of endoscopic submucosal dissection for early gastric cancer in China: a multicenter, ambispective, observational, open-cohort study

**DOI:** 10.1186/s12885-020-07312-3

**Published:** 2020-08-24

**Authors:** Zhi Zheng, Jie Yin, Ziyu Li, Yingjiang Ye, Bo Wei, Xin Wang, Yantao Tian, Mengyi Li, Qian Zhang, Na Zeng, Rui Xu, Guangyong Chen, Jie Zhang, Peng Li, Jun Cai, Hongwei Yao, Jun Zhang, Zhongtao Zhang, Shutian Zhang

**Affiliations:** 1 Department of General Surgery, Beijing Friendship Hospital, Capital Medical University, 95 Yong-an Road, Xi-Cheng District, Beijing, 100050 China; 2 Beijing Key Laboratory of Cancer Invasion and Metastasis Research, Beijing, China; 3 National Clinical Research Center for Digestive Diseases, Beijing, China; 4 Beijing Institute of Clinical Medicine, Beijing, China; 5 Department of Gastrointestinal Surgery, Beijing Cancer Hospital, Beijing, China; 6 Department of General Surgery, Peking University People’s Hospital, Beijing, China; 7 Department of General Surgery, Chinese PLA General Hospital, Beijing, China; 8 Department of General Surgery, Peking University First Hospital, Beijing, China; 9 Department of Pancreatic and Gastric Surgery, Cancer Hospital Chinese Academy of Medical Sciences, Beijing, China; 10 Clinical Epidemiology and Evidence-Based Medicine Unit, Beijing Friendship Hospital, Capital Medical University, Beijing, China; 11 Department of Pathology, Beijing Friendship Hospital, Capital Medical University, Beijing, China; 12 Department of Radiology, Beijing Friendship Hospital, Capital Medical University, Beijing, China; 13 Department of Gastroenterology, Beijing Friendship Hospital, Capital Medical University, Beijing, China

**Keywords:** Early gastric cancer, Expanded indications for ESD, Lymph node metastasis, Staging diagnosis scheme, Chinese population

## Abstract

**Background:**

The main treatment methods for early gastric cancer (EGC) include endoscopic submucosal dissection (ESD) and radical gastrectomy. However, appropriate treatment for patients who exceed the absolute indications for ESD remains unestablished. In China, evidence-based medicine for the expanding indications of ESD and accurate diagnostic staging for EGC patients are lacking. Thus, clinical studies involving Chinese patients with EGC are necessary to select appropriate treatment options and promote China’s expanded indications for ESD and diagnostic staging scheme.

**Methods:**

This is a multicenter, ambispective, observational, open-cohort study that is expected to enroll 554 patients with EGC. The study was launched in May 2018 and is scheduled to end in March 2022. All enrolled patients should meet the inclusion criteria. Case report forms and electronic data capture systems are used to obtain clinical data, which includes demographic information, results of perioperative blood- and auxiliary examinations, surgical information, results of postoperative pathology, and the outcomes of postoperative recovery and follow-up. Patients are followed up every 6 months after surgery for a minimum of 5 years. The primary endpoint is the rate of lymph node metastasis (LNM), whereas the secondary endpoints include the following: consistency, sensitivity, and specificity of the results of preoperative examinations and postoperative pathology; cut-off values for LNM; logistic regression model of expanded indications for ESD; and incidence of postoperative complications within the 30-day and 5-year relapse-free survival rates.

**Discussion:**

This study will explore and evaluate expanded indications for ESD that match the characteristics of the Chinese population in patients with EGC and will introduce a related staging procedure and examination scheme that is appropriate for China. Ethical approval was obtained from all participating centers. The findings are expected to be disseminated through publications or presentations and will facilitate clinical decision-making in EGC.

**Trial registration:**

The name of the registry is ChiCTR. It was registered on May 9, 2018, with the registration number (ChiCTR1800016084). The clinical trial was launched in May 2018 and will end in March 2022, with enrollment to be completed by December 2021. Trial status: Ongoing.

## Background

The improvement in public health awareness and the development of endoscopic technology have led to an increased detection rate and the overall incidence of early gastric cancer (EGC) [1, 2]. Statistics from the Chinese Association of Gastrointestinal Cancer Surgery show that EGC accounts for 19.5% of the overall gastric cancer cases in China [3]. Hence, scholars have begun to pay more attention to the early diagnosis and treatment of gastric cancer.

The current treatment methods for EGC mainly include endoscopic mucosal resection (EMR) or endoscopic submucosal dissection (ESD), and radical gastrectomy [4, 5]. However, the rate of lymph node metastasis (LNM) in early gastric cancer is low and, therefore, most patients undergo unnecessary or excessive lymph node dissection, which increases the surgical trauma [4]. Although EMR/ESD can be performed to completely remove the lesion, assessing the status of LNM around the stomach is currently impossible, resulting in incomplete treatment [6].

The absolute indications for ESD published in the updated Japanese gastric cancer treatment guidelines 2018 (5th edition) include the following: 1) differentiated intramucosal carcinoma with no ulcer and tumor size < 2 cm; 2) differentiated intramucosal carcinoma with no ulcer and tumor size > 2 cm; and 3) ulcerative, differentiated intramucosal carcinoma with tumor size < 3 cm that is accompanied by undifferentiated components [4]. However, controversy remains regarding the appropriate treatment for patients who exceed these absolute indications for ESD (i.e., expanded indications) [7–10]. Some studies found that ESD was suitable for some patients with EGC and expanded indications; these patients had better safety and effectiveness outcomes in the short-term after ESD [11]. Although the relative recurrence rate was increased in these patients, their long-term prognosis was better. Other studies recommend radical gastrectomy for patients with expanded indications because ESD may not meet the criteria for curative resection, and LNM may occur in the long-term [12, 13]. LNM is an independent risk factor for poor prognosis [14, 15]. Thus, a clinical trial, including patients with expanded indications for ESD, is necessary to select the treatment option that optimally balances radical tumor treatment and surgical trauma reduction.

Currently, evidence-based medicine in China supporting the use of expanded indications for ESD in patients with EGC is insufficient; hence, this multicenter, ambispective, observational, open-cohort study of Chinese patients with EGC is being conducted by the Beijing Friendship Hospital. The trial is expected to further refine the indications for ESD treatment in these patients.

## Methods/design

### Study aim

This study aims to explore and evaluate expanded indications for ESD that match the characteristics of the Chinese population in patients with EGC, and to introduce a staging procedure and examination scheme for EGC that is appropriate for China.

### Study design and setting

**Fig. 1:**
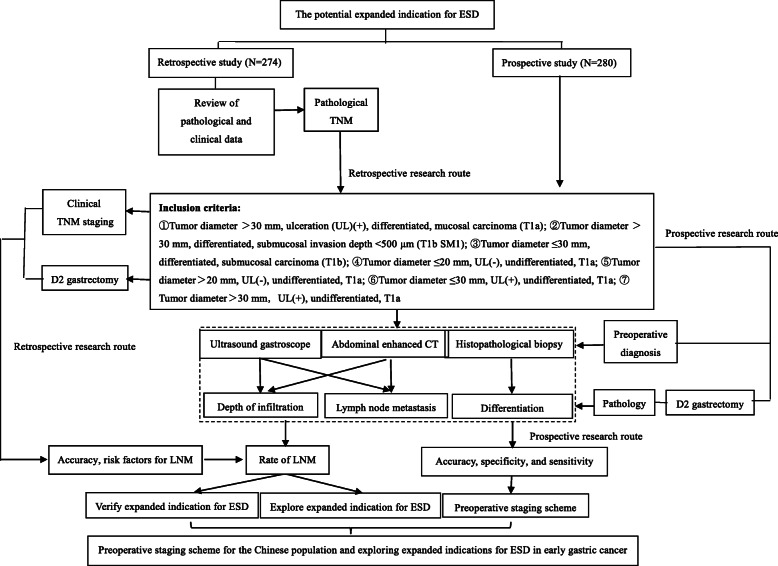
Research process and flow chart

This is a multicenter, ambispective, observational, open-cohort study. The clinical trial was launched in May 2018 and will end in March 2022, with enrollment to be completed by December 2021. Between May 2018 and December 2022, patients from Beijing Friendship Hospital, Capital Medical University, Beijing Cancer Hospital, Peking University People’s Hospital, Peking University First Hospital, Chinese PLA General Hospital, and Cancer Hospital Chinese Academy of Medical Sciences will be selected for treatment. In total, 554 patients are expected to be included in the trial. After providing written informed consent, patients will undergo a D2 gastrectomy in a non-randomized, prospective study. For the retrospective study, researchers will recruit patients from January 2008 to April 2018, and all enrolled patients must meet the inclusion criteria. The detailed research process is described in Fig. 1. The latest protocol version is version 1.0 in April 2018.

### Inclusion criteria

1. Patients aged 18–75 years (regardless of sex).
2. Patients with Eastern Cooperative Oncology Group (ECOG) score ≤ 2 points and American Society of Anesthesiologists (ASA) score ≤ 2 points who can undergo a radical D2 gastrectomy.
3. Patients without a history of gastrointestinal operation, chemotherapy, or radiotherapy.
4. Patients with normal liver, kidney, heart, lung, and bone marrow function (GPT × 10^9^/L, PLT>10^9^/L).
5. Patients who can understand and comply with the research protocol.
6. Patients who can provide written informed consent by themselves or through their legal agent.
7. Gastroscopic and pathologic diagnoses based on the following criteria: ①Tumor diameter > 30 mm, ulceration (UL)(+), and differentiated, mucosal carcinoma (T1a); ②Tumor diameter > 30 mm and differentiated, submucosal invasion depth < 500 μm (T1b SM1); ③Tumor diameter ≤ 30 mm and differentiated, submucosal carcinoma (T1b); ④Tumor diameter ≤ 20 mm, UL(−), and undifferentiated (T1a); ⑤Tumor diameter > 20 mm, UL(−), and undifferentiated (T1a); ⑥Tumor diameter ≤ 30 mm, UL(+), and undifferentiated (T1a); ⑦Tumor diameter > 30 mm, UL(+), and undifferentiated (T1a). The detailed inclusion criteria are shown in Table 1.

**Table 1:** Inclusion criteria of expanded indications for ESD in patients with EGC

	T1a				T1b			
	UL (−)		UL (+)		SM1		SM2	
	≤20 mm	> 20 mm	≤30 mm	> 30 mm	≤30 mm	> 30 mm	≤30 mm	> 30 mm
**Differentiated**	ESD	ESD	ESD	EXPANDED	EXPANDED	EXPANDED	EXPANDED	SURGERY
**Undifferentiated**	EXPANDED	EXPANDED	EXPANDED	EXPANDED	SURGERY	SURGERY	SURGERY	SURGERY

ESD, ESD absolute indication; EXPANDED, ESD expanded indication; SURGERY, surgical indication for EGC; EGC, early gastric cancer; ESD, endoscopic submucosal dissection

### Exclusion criteria

1. Patients with a contraindication for gastroscopy.
2. Patients with uncontrollable diseases, such as coagulation disorders, epilepsy, central nervous system diseases or mental disorders, cardiopulmonary insufficiency, unstable angina, myocardial infarction, a cerebrovascular accident that occurred within 6 months, and other surgical contraindications.
3. Patients who cannot undergo general anesthesia or surgical treatment because of conditions related to other organs, or patients who are unwilling to undergo surgery.
4. Patients with gastric stump cancer, recurrent gastric cancer, multiple primary malignant tumors in the abdominopelvic cavity, or a history of other malignant tumors within the previous 5 years.
5. Patients who are pregnant or lactating.
6. Patients who are participating in other clinical trials.

### Elimination criteria

1. A tumor with distant metastasis that is observed during the operation and postoperatively confirmed as advanced gastric cancer (pT_2_N_0_-_3_ M_0–1_) or non-gastric cancer by pathologic examination.
2. Patients who do not follow the research plan and receive other anti-tumor treatments during the observation period.
3. Patients with incomplete clinical data obtained after enrollment, which cannot be used in future statistical analysis.
4. Patients who do not comply with the research protocol for treatment.
5. Study termination as deemed necessary by researchers for the benefit of the patient.

### Primary endpoint

The primary endpoint of this study is the rate of LNM. The scope of potential expanded indications for ESD is explored and evaluated based on the presence of LNM by histopathology and the positive rate of LNM with expanded indications for ESD.

### Secondary endpoints

1. Accuracy, specificity, and sensitivity are evaluated using the following gold standards: postoperative histopathology of ultrasonic gastroscopy for tumor invasion depth, abdominal and pelvic enhanced computed tomography (CT) scan for LNM, pathological biopsy for tumor differentiation, and auxiliary examination for TNM staging.
2. Cut-off values for LNM: A receiver operating characteristic curve is drawn for the clinical characteristics under different LNM rates to obtain the cut-off values.
3. Logistic regression model: To explore the corresponding logistic regression model, postoperative histopathological examination for LNM, depth of infiltration, and degree of differentiation are evaluated in the univariate analysis. Variables with statistical significance in the univariate analysis are examined in the multivariate analysis.
4. Complications: The incidence of postoperative complications within the last 30 days, including anastomotic fistula, pancreatic fistula, intestinal obstruction, delayed bleeding, and incision-related complications, are recorded. Postoperative complications classified as higher than Grade II, according to the Clavien–Dindo classification are regarded as clinically significant (Tables 2), [16].
5. Five-year relapse-free survival rate: The time interval from the date of operation to the detection of tumor recurrence within 5 years is recorded.

**Table 2:** Clavien–Dindo classification

Grade	Definition
I	Any complication that deviates from the natural course of the operation; treatments include antiemetic, antipyretic, analgesic, and diuretic drugs; infusion; physical therapy; as well as bedside debridement of incision infection.
II	Medications other than those permitted for grade I complications are required, including blood transfusion and total parenteral nutrition support.
III	Surgical and endoscopic interventions and radiotherapy are required.
IIIa	No general anesthesia is required.
IIIb	General anesthesia is needed.
IV	Life-threatening complications requiring intensive care.
IVa	Single-organ dysfunction
IVb	Multi-organ dysfunction
V	Death

### Participating surgeons

Studies have found that surgeon proficiency is significantly related to rates of postoperative complications, mortality, and the presence of a residual tumor. Concurrently, a study analyzed surgeons’ operation and found that a surgeon had completed the surgery learning curve after performing 90–100 operations and could independently handle emergencies during surgery [17, 18]. The length of surgery, blood loss, and incidence of intraoperative and postoperative complications similarly decreased. Therefore, all surgeons involved in this study will have completed at least 100 gastrectomy surgeries to ensure the quality of treatment for the enrolled patients. Similarly, our research team includes several experienced gastrointestinal surgeons who are available to perform surgeries for enrolled patients.

### Informed consent

According to the requirements of the ethics committee, all enrolled patients must sign an informed consent document. Informed consent forms are provided to the patients before their enrollment in the prospective study; the consent forms include information on the purpose and significance of the study, the benefits and possible risks of participating in the study, and the confidentiality of the study. Enrolled patients have the opportunity to ask questions and receive answers. The requirement for informed consent is waived due to the retrospective nature of study.

### Interventions

#### D2 gastrectomy

Under the requirement of the guideline for laparoscopic gastrectomy for gastric cancer (2016 edition) [19], laparoscopic radical gastrectomy is performed in patients. A preoperative abdominal enhanced CT scan is performed to evaluate the lesion site, tumor size, and LNM. Preoperative endoscopic injection positioning is performed with dye, or intraoperative endoscopic positioning is used to determine the tumor location to ensure safe margins. The scope of lymph node dissection (LND) is according to the Japanese gastric cancer treatment guidelines 2018 (5th edition) [4]. The scope of LND of D2 distal gastrectomy should include the No. 1, 3, 4sb, 4d, 5, 6, 7, 8a, 9, 11p, and 12a lymph nodes. D2 proximal gastrectomy should include the 1, 2, 3a, 4sa, 4sb, 7, 8a, 9, 11p, and 12a lymph nodes, and D2 total gastrectomy should include the 1, 2, 3, 4sa, 4sb, 4d, 5, 6, 7, 8a, 9, 11p, 11d, and 12a lymph nodes.

#### Perioperative treatment for enrolled patients

For patients who underwent radical surgery, clinicians will provide symptom-based treatment, such as antibiotics, proton pump inhibitors, analgesics, octreotide, total parenteral nutrition support, and blood products, according to the patient’s recovery. Routine postoperative blood tests, biochemistry tests, blood amylase, and abdominal drainage fluid amylase will be reviewed regularly to monitor for anastomotic leakage, delayed bleeding, and pancreatic leakage. There will be a regular abdominal ultrasound examination to monitor for peritoneal effusion. If necessary, drainage by abdominal puncture will be performed. After return to a normal diet, patients with LNM or undifferentiated tumors will receive adjuvant chemotherapy 3–4 weeks after surgery.

#### Data collection

A uniform case report form (CRF) was designed, and the electronic data capture (EDC) system was already established before the commencement of the clinical trial. All data are provided on the CRF. Subsequently, the clinical research coordinator (CRC) enters the data into the EDC system promptly (https://edc-cloud.medsci.cn/#/login). Every month, clinical research associates monitor the electronic database to ensure data quality.

The CRF includes the following data: 1) Demographic information: sex, age, length of hospital stay, body mass index, family disease history, concomitant disease, ECOG score, nutrition risk screening 2002 (NRS 2002) score, and venous thromboembolism score; 2) Perioperative laboratory tests: results of routine blood tests, biochemical investigations, and tumor marker evaluations, including CA19–9, CA125, AFP, CEA, and CA724; 3) Auxiliary examination: all the enrolled patients undergo an abdominal enhanced CT scan and low-dose plain CT scan of the chest, a positron emission tomography (PET)/CT examination when necessary, and an ultrasound gastroscopy or magnified gastroscopy; 4) Surgical information: ASA score, operation date, operation time, blood loss, surgical methods, degree of lymph node dissection, and intraoperative complications; 5) Postoperative pathology: the number of tumor lesions, tumor size, tumor location, surgical margin, gross type of tumor, histopathological type, Lauren type, depth of tumor invasion, number of lymph nodes dissected, and total number of LNM; and 6) Postoperative recovery outcomes: total hospitalization cost, postoperative complications and death, and recovery time of intestinal function (Table 3).

**Table 3:** Checklist for the collection of necessary clinical data and follow-up scheme of enrolled patients with EGC

	Baseline information		Follow-up after operation													
	Preoperation	Operation	POD 1	POD 3	POD 7	POD 30	6 months	1 year	18 months	2 years	30 months	3 years	42 months	4 years	54 months	5 years
**Informed consent**	×															
**Demographic information**	×															
**Laboratory tests**	×		×	×	×	×	×	×	×	×	×	×	×	×	×	×
**Chest CT scan**	×							×		×		×		×		×
**Abdominal CT scan**	×						×	×	×	×	×	×	×	×	×	×
**Gastroscopy**	×							×		×		×		×		×
**Abdominal ultrasound**	×						×	×	×	×	×	×	×	×	×	×
**Physical examination**	×						×	×	×	×	×	×	×	×	×	×
**Surgical information**		×														
**Pathology**		×														
**Postoperative recovery outcomes**			×	×	×	×										

× indicates the need to collect the clinical data

POD, postoperative day; EGC, early gastric cancer; Chest CT scan, chest computed tomography scan; Abdominal CT scan, abdominal computed tomography scan

#### Follow-up

The postoperative follow-up is performed by a dedicated investigator. The patients are followed up every 6 months after discharge using outpatient visits, telephone, or mail. During the follow-up period, the patients undergo physical examinations, laboratory tests, a gastroscopy, a chest and abdominal CT scan, or an abdominal ultrasound assessment. The laboratory tests include routine blood examination, blood biochemistry, and tumor marker evaluation that includes CA19–9, CA125, AFP, CEA, and CA724. For patients that develop postoperative tumor recurrence or distant metastasis, a further detailed evaluation, including abdominal magnetic resonance imaging or PET/CT, will be performed to determine the feasibility of surgical resection. Each patient will be followed for at least 5 years or until a loss to follow-up. The detailed follow-up schedule is shown in Table 3.

#### Adverse events

All serious adverse events (SAEs) occurring between the signing of the informed consent and the completion of the trial need to be recorded. SAEs are defined as any injury related (or not) to the expected outcome of the operation. The trial includes an independent data monitoring committee that will review the ongoing safety data in an unblinded manner under the Standard Operation Procedures for Clinical Trials, Japan Medical Association. All the patients will undergo the best treatments for curing complications.

### Quality control

#### Quality control of pathology

**Fig. 2:**
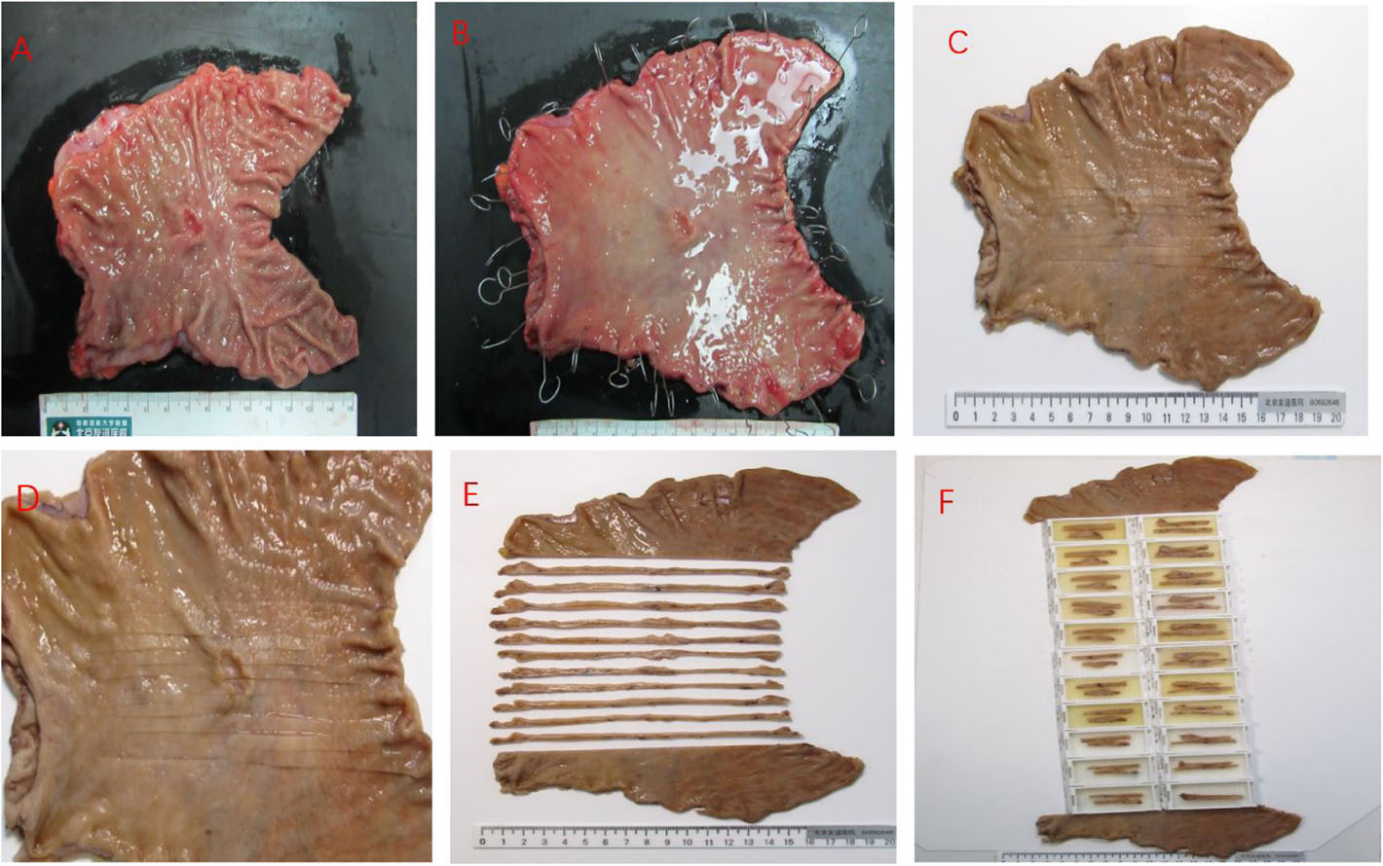
Standardized procedure for sampling of gastric specimen by surgical resection: **a** incision of the specimen along the greater curvature of the stomach; **b** the resected specimen was pinned out with fine needles on a rubber plate; **c** the resected specimen was soaked in a volume of 4% neutral buffered formaldehyde 6–8 times that of the specimen for 12–48 h; **d** and **e** the whole of the gastric wall tissue was sliced in parallel at a distance of 4–5 mm; **f** the sliced tissues were placed in the cassettes for subsequent dehydration, embedding, and sectioning for histological evaluation

The specimen collectors have sampling experience or are guided by an experienced pathologist. The procedures for handling the resected specimens are as follows: First, the pathologist incises the stomach along the greater curvature. Second, the specimen is fully stretched to maintain the original shape, and the stomach is placed on a flat rubber plate with the mucosal side up and pinned at the edge with stainless steel pins. At the edge of the specimen, the entire gastric wall, especially the muscularis, is pulled outward by the needle with a uniform force to extend the mucosa, which is subsequently fixed on the rubber plate to fully expose the lesions of the mucosal surface. Third, the specimen collection is performed within 30 min after the tissue is isolated. Thereafter, we completely immerse the specimen in a volume of 10% neutral buffered formalin 6–8 times that of the specimen for 12–48 h. The tumor lesions are afterwards sectioned along the vertical direction of the tangent line near the cutting edge. Subsequently, the whole gastric wall tissue is cut in parallel at a distance of 4–5 mm, and all tissues are collected for examination. Finally, we dehydrate, embed, and section the tissue for microscopic observation. The detailed procedure is illustrated in Fig. 2.

Moreover, we have developed a standardized pathology report template to homogenize the data from all research centers. The pathology report includes the macroscopic description, histological diagnosis, Lauren type, depth of invasion, vascular-lymphatic infiltration, surgical margin, LNM, pathological tumor stage, immunohistochemical staining, and gene detection.

#### Quality control of gastroscopy

We have developed a gastroscopy report template, which helps to unify the research data; the report includes a general description of the stomach. We need to obtain 4–8 images of the different parts of the stomach. Furthermore, the report describes the number of lesions, tumor location, tumor size, gross type of tumor, tumor distance from the dentate line, and tumor distance from the pyloric ring. If patients undergo ultrasound gastroscopy, the report should equally describe the depth of invasion and the occurrence of LNM around the stomach.

#### Quality control of radiographic examination

An abdominal and pelvic enhanced CT scan is important for clinical diagnosis and tumor staging. We ensure that the following items are determined with the CT scan: tumor location, tumor size, presence or absence of abdominal effusion, other abdominal organ metastasis, non-local LNM, and abnormal variation of peripheral gastric blood vessels; these aspects are included in the imaging report template.

#### Research supervision committee

We ensure that members of the research supervision committee of this trial have a clear division of labor and good cooperation. The supervision committee consists of data managers, data inspectors, and methodological teams. Each part of this clinical trial has a standard operating procedure to ensure the homogeneity of the research. Moreover, specialized personnel is engaged in data collection, data entry, data inspection, data cleaning, and follow-up.

### Statistical analysis

SPSS 21.0 (IBM Corp., Armonk, NY, USA) statistical software is used for the statistical analysis. The quantitative variables are described as the mean ± standard deviation and are tested by univariate analysis of variance for normal distribution; on the contrary, they are described as the median (interquartile range) and tested by the Kruskal-Wallis H test for non-normal distribution. The categorical variables are described as frequency (N) with the percentage (%) and tested by χ^2^ test, corrective χ^2^ test, or Fisher’s exact test. Ranked data are expressed as frequency and percentages and are compared using the Kruskal-Wallis H test. Sensitivity and specificity are calculated to evaluate the diagnostic performance. The Kaplan-Meier method is used to draw survival curves, and the survival of multiple groups are compared by the log-rank test. The univariate and multivariate Cox proportional hazards models are used to evaluate the hazard ratios for adverse outcomes. All statistical tests are two-sided, and a *P*-value < 0.05 is considered statistically significant.

### Determination of sample size

The sample size was calculated by PASS 11.0 (NCSS Statistical and Data Analysis, USA) software and was estimated based on the preliminary research results of the Japanese clinical trial. According to previous studies with a single group design, the rate of LNM with expanded indications for ESD was 3% [20, 21] with an allowable error of 1.5%. Based on a one-sided test with an α value equal to 0.025, the estimated total sample size required for the trial is at least 497 patients. The withdrawal rate was assumed to be 20% during the follow-up. Therefore, the total sample size that is needed for this clinical study is approximately 554 patients, of which 274 and 280 are enrolled in a prospective- and retrospective study, respectively.

### Patient and public involvement

Patients and the public were not involved in the design, the recruitment, or the conduct of the trial. The sponsor played an important role in study design, collection, management, analysis and interpretation of data, writing of the report, and the decision to submit the report for publication. They had the ultimate authority regarding any activity in the trial.

## Discussion

The optimal treatment for EGC should balance radical tumor treatment and surgical trauma reduction. However, controversy remains surrounding how to choose an appropriate treatment in patients with EGC who exceed the absolute indications for ESD. The primary endpoint of this study is the rate of LNM. The scope of potential expanded indications for ESD will be explored and evaluated based on the presence of LNM by histopathology and the positive rate of LNM with expanded indications for ESD. The purpose of this study is to explore and evaluate expanded indications for ESD that match the characteristics of the Chinese population in patients with EGC and to introduce a staging diagnosis and examination scheme for EGC that is appropriate for China. To the best of our knowledge, this study is the first clinical trial to focus on Chinese patients with EGC, and the results could facilitate clinical decision-making.

Despite decades of research on the treatment of EGC, numerous problems remain unsolved. Regarding preoperative evaluation, Choi et al. showed that the overall accuracy of endoscopic ultrasonography in T staging of gastric cancer is 78% and only 72% in T1b submucosal staging. Thus, determining the depth of tumor submucosal infiltration remains difficult, which may result in unnecessary surgical trauma to some patients [22]. Similarly, Choi et al. reported that the tumor diameter measured under the endoscope was significantly smaller than that measured pathologically. Although the size deviation in 80% of tumor lesions was < 0.4 cm, the difference suggests that preoperative gastroscopy may underestimate the tumor diameter [23]. Consequently, ESD performed in patients with expanded indications may result in non-curative resection of the tumor and, thereby, affect the prognosis of patients.

Most of the endoscopic treatments of EGC worldwide are based on the Japanese gastric cancer treatment guidelines 2018 (5th edition) [4]. Most of the existing research data on the absolute indications for ESD are from Japan and South Korea. Some studies have found that performing ESD for expanded indications can achieve a similar effect on LNM rate and patient prognosis as performing ESD for absolute indications [24, 25]. Therefore, a refinement of the expanded indications for ESD is imperative to guide clinical practice. Additionally, most of the studies on ESD or gastrectomy are retrospective in nature, and the influence of preoperative examination, surgeon, and surgical procedure may lead to bias; moreover, a detailed subgroup analysis was lacking. Further, prospective, multicenter, large-sample studies on radical gastrectomy in the Chinese population to verify the feasibility of endoscopic indications for EGC or adjust the existing endoscopic indications for EGC are limited. Hence, conducting this study to explore the expanded indications for EGC is necessary.

This study has some limitations. First, this study includes only a Chinese sample; therefore, further studies that include other populations are warranted. Second, this is a multicenter, ambispective, observational, open-cohort study; the quality of evidence may be lower than a large-sample, multicenter, randomized controlled study. Nevertheless, we will improve the research protocol further, by including more research centers and patients to provide a more precise evaluation of expanded indications for ESD and staging scheme for EGC.

### Ethics and dissemination

This protocol has been reviewed and approved by the Ethics Committee of Beijing Friendship Hospital, Capital Medical University (2018-P2–015-02). The medical teams involved in this study have well-developed and standardized surgical techniques, and extensive experience, which reduces the potential risks associated with surgery and ensures the correct implementation of this study. In the event of complications, the medical teams have standardized measures to maximize the patient’s safety.

During the study, all personal data, such as name and sex, will be replaced with statistical codes or numbers and will be kept strictly confidential. All clinical data will be analyzed anonymously through the CRF and EDC systems to protect the privacy of the patients. Similarly, we plan to publish the results of this study in 2023 once all data have been obtained and evaluated. However, the article will not disclose any personal information.

## Data Availability

We will transfer the CRFs to the EDC (https://edc-cloud.medsci.cn/#/login), which will be stored in a hard disk and cloud system. Detailed results will be openly shared after study completion.

## Abbreviations

EGC: Early gastric cancer
ESD: Endoscopic submucosal dissection
LNM: Lymph node metastasis
EMR: Endoscopic mucosal resection
ECOG: Eastern Cooperative Oncology Group
ASA: American Society of Anesthesiologists
UL: Ulceration
CT: Computed tomography
CRF: Case report form
EDC: Electronic data capture
CRC: Clinical research coordinator
NRS2002: Nutrition risk screening 2002
POD: Postoperative day
